# Characterization of the Ectodomain of the Envelope Protein of Dengue Virus Type 4: Expression, Membrane Association, Secretion and Particle Formation in the Absence of Precursor Membrane Protein

**DOI:** 10.1371/journal.pone.0100641

**Published:** 2014-06-20

**Authors:** Szu-Chia Hsieh, Wen-Yang Tsai, Vivek R. Nerurkar, Wei-Kung Wang

**Affiliations:** 1 Department of Tropical Medicine, Medical Microbiology and Pharmacology, John A. Burns School of Medicine, University of Hawaii at Manoa, Honolulu, Hawaii, United States of America; 2 Pacific Center for Emerging Infectious Diseases Research, John A. Burns School of Medicine, University of Hawaii at Manoa, Honolulu, Hawaii, United States of America; Utah State University, United States of America

## Abstract

**Background:**

The envelope (E) of dengue virus (DENV) is the major target of neutralizing antibodies and vaccine development. After biosynthesis E protein forms a heterodimer with precursor membrane (prM) protein. Recent reports of infection enhancement by anti-prM monoclonal antibodies (mAbs) suggest anti-prM responses could be potentially harmful. Previously, we studied a series of C-terminal truncation constructs expressing DENV type 4 prM/E or E proteins and found the ectodomain of E protein alone could be recognized by all 12 mAbs tested, suggesting E protein ectodomain as a potential subunit immunogen without inducing anti-prM response. The characteristics of DENV E protein ectodomain in the absence of prM protein remains largely unknown.

**Methodology/Principal Findings:**

In this study, we investigated the expression, membrane association, glycosylation pattern, secretion and particle formation of E protein ectodomain of DENV4 in the presence or absence of prM protein. E protein ectodomain associated with membrane in or beyond trans-Golgi and contained primarily complex glycans, whereas full-length E protein associated with ER membrane and contained high mannose glycans. In the absence of prM protein, E protein ectodomain can secrete as well as form particles of approximately 49 nm in diameter, as revealed by sucrose gradient ultracentrifugation with or without detergent and electron microscopy. Mutational analysis revealed that the secretion of E protein ectodomain was affected by N-linked glycosylation and could be restored by treatment with ammonia chloride.

**Conclusions/Significance:**

Considering the enhancement of DENV infectivity by anti-prM antibodies, our findings provide new insights into the expression and secretion of E protein ectodomain in the absence of prM protein and contribute to future subunit vaccine design.

## Introduction

The four serotypes of dengue virus (DENV1, DENV2, DENV3, and DENV4), belonging to the genus *Flavivirus* in the family *Flaviviridae*, cause the most common and important arboviral diseases in humans in the tropical and subtropical areas [1]–[3]. While most DENV infections are asymptomatic or result in a self-limited disease, known as dengue fever, some may develop severe and potentially life-threatening disease, dengue hemorrhagic fever/dengue shock syndrome. Despite numerous efforts to develop therapeutic or prophylactic interventions, there is no licensed antiviral or DENV vaccine currently available [1]–[3]. DENV is a positive-sense, single-stranded RNA virus containing a genome of approximately 10.6 kb. Flanked by the 5′ and 3′ untranslated regions, the single open reading frame encodes a polyprotein precursor, which is cleaved by cellular and viral protease into three structural proteins, capsid, precursor membrane (prM) and envelope (E), and seven nonstructural proteins [4].

DENV enters the cell through receptor-mediated endocytosis [4]–[7]. After uncoating, translation and genome replication, assembly of viral particles occurs in the membranes derived from endoplasmic reticulum (ER). Immature virions containing prM and E proteins bud into the lumen of ER and transport through the secretary pathway [4], [8]–[10]. Following cleavage of prM protein on immature virions by furin or furin-like protease in the trans-Golgi, mature virions are generated and released from cells, though the cleavage was not efficient for DENV [11]–[15]. In addition to mature and immature virions, small and slowly sedimenting subviral particles are formed during flaviviral replication [4], [16]. Co-expression of prM and E proteins can produce recombinant virus-like particles (VLPs), which are similar to the infectious virions in the biophysical and antigenic properties [17]–[19].

**Figure 1:**
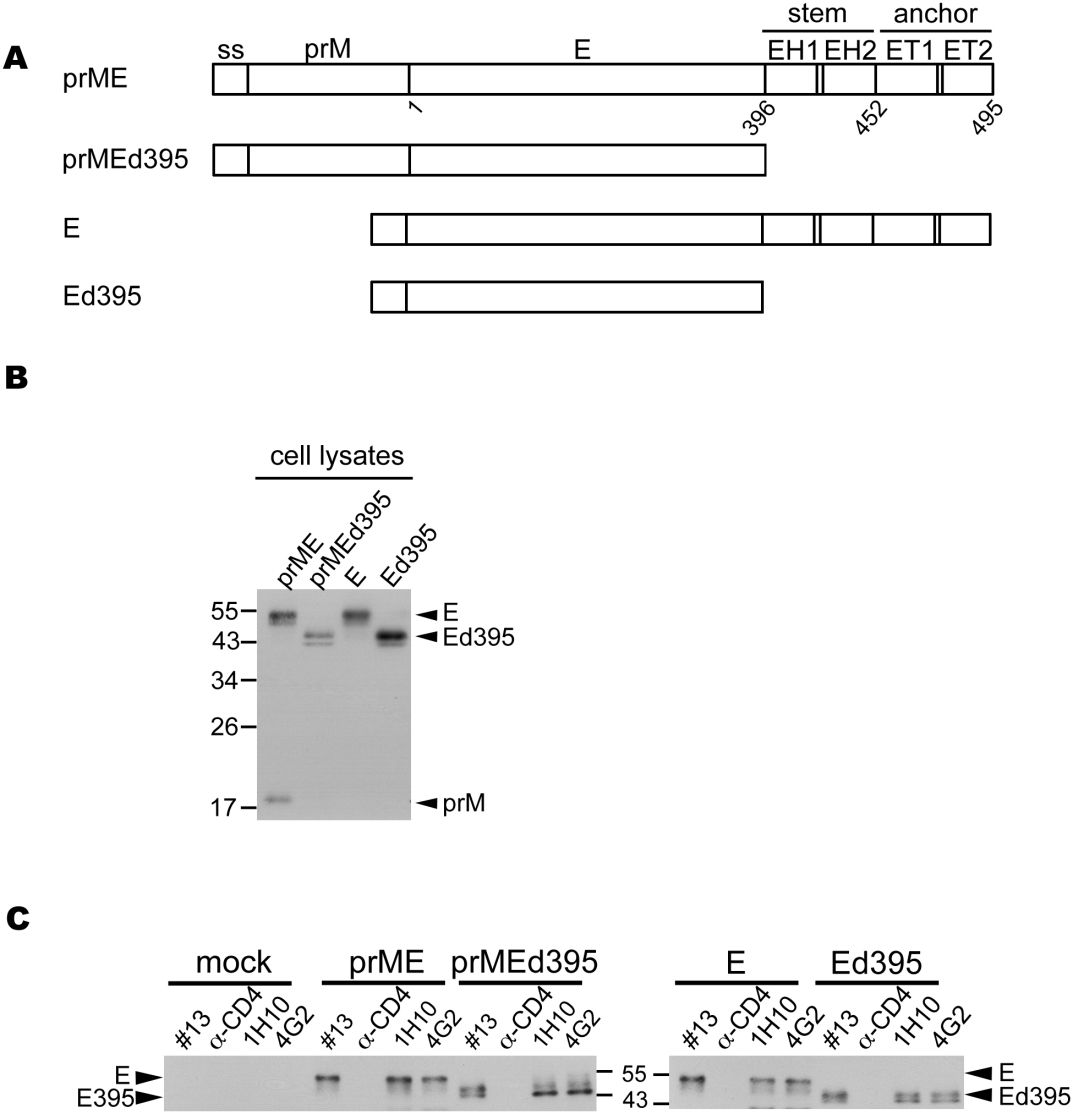
Expression of E protein and E protein ectodomain in the presence or absence of prM protein. (A) Schematic drawing of the DENV4 prME, prMEd395, E and Ed395 constructs. The C-terminus of E protein contains two α-helical domains (EH1 and EH2) in the stem region, followed by two transmembrane domains (ET1 and ET2) in the anchor region [24]. ss, signal sequence. (B, C) At 48 h, lysates of 293T cells transfected with each of the 4 constructs were subjected to Western blot analysis (B) using a dengue-immune serum [39] or immunoprecipitation (C) using two mouse anti-E mAbs (4G2 and 1H10-6-7) and a dengue-immune human serum (#13), followed by Western blot analysis [38]. Anti-CD4 mAb was used a negative control. One representative experiment of two was shown. The size of molecular weight markers is shown in kDa.

The E protein plays an important role in virus entry, and is the major target of neutralizing antibodies and vaccine development [20], [21]. Based on X-ray crystallographic studies, the N-terminal ectodomain of E protein comprises of three domains (domains I, II and III) [22], [23]. At the C-terminus of E protein, there are two α-helices (EH1 and EH2) in the stem region and two transmembrane domains (ET1 and ET2) in the anchor region [24] (Figure 1A). Previous studies of the tick-borne encephalitis virus (TBEV) have shown both ET2 and ET1 were required for the assembly of E protein into VLPs [19], [25], [26]. A study of the yellow fever virus (YFV) reported that transmembrane domains of prM and E proteins were involved in the formation of VLPs [27].

After biosynthesis in the rough ER, prM and E proteins form a heterodimer, which was reported to be important for assembly of VLPs [25], [26]. The prM protein has been shown to serve as a chaperon of E protein [28], [29] and to prevent E protein from premature fusion within acidic compartments along the secretary pathway [30], [31]. While a previous study reported a protective role of anti-prM antibodies against DENV infection in mice [32], recent studies showed that anti-prM mAbs neutralize DENV poorly and potently enhanced the infection of immature DENV particles by antibody-dependent enhancement (ADE) [33]–[35], suggesting anti-prM responses should be minimized in dengue vaccines [35]. Due to the inefficient cleavage of prM protein on DENV virions [11]–[15], the presence of prM protein in DENV vaccine preparation (live attenuated or killed virus vaccine) could represent enhancing epitopes and be potentially harmful [35].

Previously, we studied a series of C-terminal truncation constructs expressing DENV4 prM/E or E proteins, and found that the ectodomain of E protein alone can be recognized by all 12 mouse and human mAbs tested [36]. Considering the effect of ADE mediated by anti-prM antibodies, this finding suggests that E protein ectodomain could be a potential subunit immunogen that preserves the conformation of E protein without inducing anti-prM response. The expression and characteristics of DENV E protein ectodomain in the absence of prM protein remains largely unknown. In this study, we investigated the expression, membrane association, glycosylation pattern, secretion and particle formation of E protein ectodomain of DENV4 in the presence or absence of prM protein. We found that E protein ectodomain associated with membrane in or beyond trans-Golgi and contained primarily complex glycans, whereas full-length E protein associated with ER membrane and contained high mannose glycans. In the absence of prM protein, the ectodomain of E protein can secrete as well as form particles of approximately 49 nm in diameter. Mutational analysis revealed that the secretion of E protein ectodomain was affected by N-linked glycosylation and could be restored by treatment with ammonia chloride.

## Methods

### Plasmid Constructs

The prM/E expression construct of DENV4 (pCB-D4, designated as prME in this study), prMEd395 and Ed395 constructs were described previously [37]. To construct E alone, PCR fragment containing signal sequence and E protein amino acid 1 to 495 was amplified using the primer pair (d4KpnSSEA and d4E495BNotI) and prME as template. After digestion with KpnI and NotI, the PCR product was cloned into respective sites of pCB vector. All constructs were confirmed by sequencing the entire inserts to rule out second site mutations. The sequences of all primers used will be provided upon request.

### Cell Lysates, VLPs and Western Blot Analysis

293T cells prepared in a 10 cm-culture dish at 5×10^5^ cells per dish one day before were transfected with 10 µg of plasmid DNA by calcium phosphate method [38]. For ammonia chloride treatment, culture supernatants were replaced with fresh medium containing 20 mM HN_4_Cl at 18 h post-transfection. Culture supernatants were collected at 48 h post-transfection (see below); cells were washed with PBS and treated with 1% NP40 lysis buffer, followed by centrifugation at 20,000×g at 4°C for 30 min to obtain cell lystates [36]. Culture supernatants were clarified by centrifugation at 1,250×g for 20 min, filtered through a 0.22 µm pore-sized membrane (Millipore), layered over a 20% sucrose buffer, and ultracentrifuged at 65,000×g at 4°C for 5 h to obtain pellets, which were resuspended in 60 µl TNE buffer [38]. For Western blot analysis, cell lysates or pellets were added to non-reducing buffer and subjected to 12% polyacrylamide gel electrophoresis (PAGE) [36], followed by transfer to nitrocellulose membrane, blocking and incubation with primary antibody (human serum from confirmed dengue case, mouse anti-β actin [Sigma] or anti-calnexin mAb E-10 [Santa Cruz]) and secondary antibody [39]. After final washing, the signals were detected by enhanced chemiluminescence reagents (Perkin Elmer life sciences) [38].

### Immunoprecipitation

Protein A sepharose beads (Amersham Biosciences, HK) was incubated with anti-CD4 mAb (SK3), anti-E mouse mAbs (4G2, 1H10-6-7) and human serum at 4°C for 6 h and then incubated with cell lysates or culture supernatants at 4°C overnight. After washing with 1% NP40 buffer 4 times, the beads were mixed with 15 µl 2X sample buffer [36], heated at 95°C for 5 min, and centrifuged at 20,000×g for 1 min. The solubilized fraction was subjected to 12% PAGE.

### Sedimentation Analysis

Plasmid DNA was transfected to 293T cells prepared in a 10-cm culture dish by calcium phosphate method. At 48 h post-transfection, cells were washed, resuspended in PBS, treated with 1% Triton X-100 on ice for 60 min, and then loaded into a 5 to 20% (wt/wt) sucrose gradient made with gradient buffer (50 mM Tris-HCl [pH 8.0], 150 mM NaCl, 2 mM EDTA, 0.5% Triton X-100, 1 mM phenylmethylsulfonyl fluoride). The gradient was ultracentrifuged at 247,606×g (SW41 rotor, Beckman) at 15°C for 22 h, and each of the 13 fractions was collected and subjected to Western blot analysis [38].

### Membrane Flotation Assay

293T cells (1×10^5^ cells) prepared in 10 cm dish were transfected with 10 µg DNA by calcium phosphate method. The transfected cells were washed with PBS, lysed in 1 ml of hypotonic buffer (10 mM Tris-HCl [pH 7.5], 10 mM KCl, 5 mM MaCl_2_) and passed through a 25-gauge needle 20 times. After centrifugation at 1,000×g at 4°C for 5 min, 0.8 ml postnuclear supernatants were mixed with 3 ml of 72% sucrose in low-salt buffer (LSB, 50 mM Tris-HCl [pH 7.5], 25 mM KCl and 5 mM MgCl_2_), overlaid with 4 ml of 55% sucrose in LSB and then 1.5 ml of 10% sucrose in LSB, followed by ultracentrifugation at 247,606×g (SW41 rotor, Beckman) at 4°C for 14 h [40]. A total of 9 fractions were collected; each fraction was subjected to Western blot analysis.

### Subcellular Fractionation and Enzyme Digestion

293T cells transfected with plasmid DNA were washed 3 times with PBS at 48 h, resuspended in modified buffer B, and subjected to freeze-thaw cycle 8 times as described previously [38]. After clearing the nuclei and debris by centrifugation at 1,000×g for 5 min, the membrane fraction was obtained by centrifugation at 20,000×g for 30 min at 4°C. The resulting supernatants were layered over a 20% sucrose buffer and ultracentrifuged at 246,000×g (SW55 rotor, Beckman) at 4°C for 1 h to obtain the pellets of soluble fraction, which were resuspended in 30 µl TNE buffer [38]. Membrane fraction and pellets of soluble fraction were subjected to Western blot analysis. Aliquots from above were treated with endo-β-N-acetylglucosaminidase H (endo H) or peptide N-glycosidase F (PNGase F) at 37°C for 1 h according to the manufacture’s instructions (New England Biolabs), and subjected to Western blot analysis.

### Separation of VLPs by Sucrose Gradient

An aliquot of the pellets ultracentrifuged by 20% sucrose cushion was treated with or without Triton X-100 on ice for 60 min and overlaid with 15–55% sucrose gradient in TNE buffer, followed by ultracentrifugation at 107,170×g (SW41 rotor, Beckman) at 4°C for 16 h. A total of 9 fractions was collected; each fraction was subjected to Western blot analysis. The density of each fraction was determined by a refractometer.

### Electron Microscopy (EM)

An aliquot of VLPs was absorbed to a glow-discharged 300-mesh formvar/carbon film, stained with 2% phophotungstic acid (PTA) (Electron Microscopy Sciences), and examined by transmission electron microscopy (HT7700, Hitachi). For immunogold labeling, the pellets overlaid with 5–20% sucrose gradient in PBS, followed by ultracentrifugation at 247,606×g (SW41 rotor, Beckman) and 4°C for 90 min. Each fraction was first diluted three folds in PBS, then ultracentrifuged at 65,000×g (SW41 rotor, Beckman) and 4°C for 5 h and resuspended in PBS [41]. Gradient-purified particles were absorbed to a glow-discharged 300-mesh nickel grid for 10 min. After blocking with 3% BSA in Tris-buffer saline, grids were incubated for 1 h at room temperature with anti-E mAbs (1H10-6-7) in Tris-buffered saline. Grids were then washed three times with Tris-buffered saline and incubated at room temperature for 1 h with goat anti-mouse antibody conjugated with 6 nm gold particles (Electron Microscopy Sciences) and diluted 1: 20 in Tris-buffered saline. Grids were washed with deionized water and stained as described above.

### Capture ELISA

Flat-bottom 96 well plate was coated with mouse mAb (1H10-6-7) against DENV4 E protein at 4°C overnight, followed by blocking with 1% BSA in PBS for 1 h and adding culture supernatants or recombinant DENV4 E protein, anti-E human mAbs and anti-human IgG conjugated with HRP each at 37°C for 1 h, and adding TMB substrate and stop solution [42]. Comparable amounts of recombinant DENV4 E, prMEd395 and Ed395 added were confirmed by polyclonal human serum. The absorbance at wavelength of 450 nm (OD450) with reference wave length of 650 nm was read.

## Results

### The Ectodomain of E Protein Alone Expresses Well and Forms Oligomers in Cells

To investigate the expression, membrane association and glycosylation pattern of the ectodomain of DENV4 E protein in the presence or absence of prM protein in comparison with those of the full-length E protein, we generated four constructs encoding full-length E protein and E protein ectodomain with or without prM protein (Figure 1A). After transfection to 293T cells, Western blot analysis of cell lysates revealed comparable amounts of E protein expressed by these four constructs (Figure 1B). Immunoprecipitation of cell lysates using human serum and two anti-E mAbs (1H10-6-7 and 4G2, which recognize domains III and II, respectively) revealed that the amount of E protein expressed by prMEd395, E or Ed395 construct was generally comparable to that by prME construct (Figure 1C), suggesting that in the absence of prM protein, E protein and E protein ectodomain preserve the epitopes recognized by these two mAbs and human serum.

**Figure 2:**
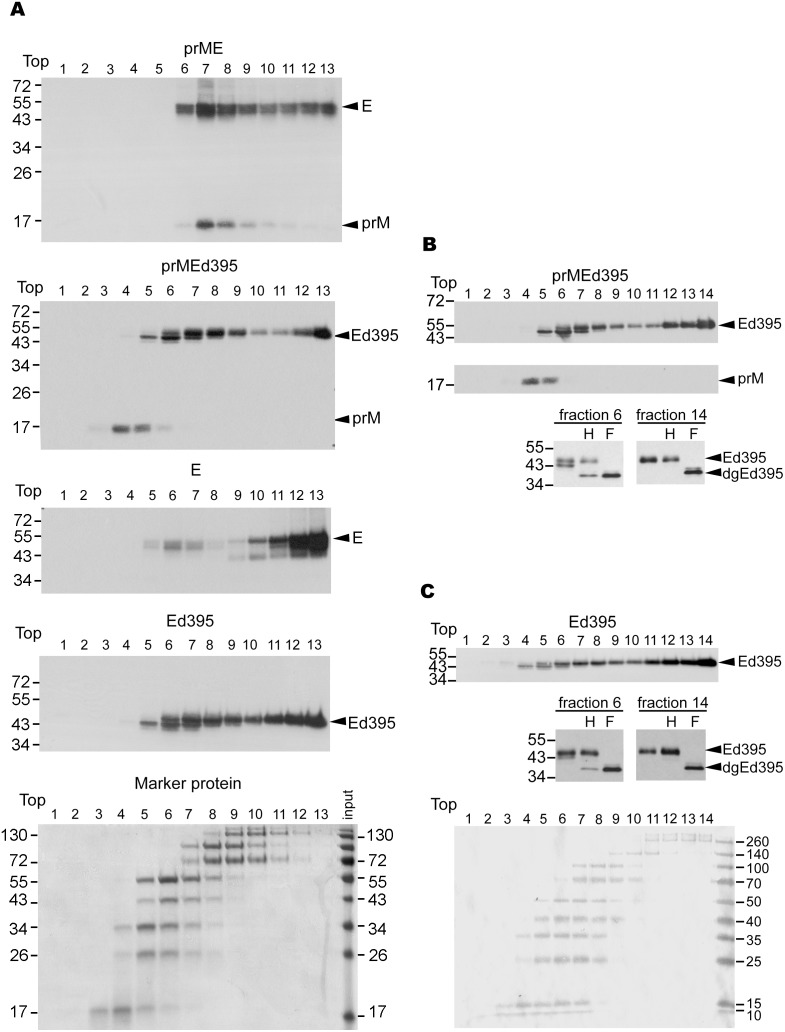
Oligomeric status of E protein and E protein ectodomain in cells by sucrose gradient sedimentation analysis. (A) At 48 h, 293T cells transfected with prME, prMEd395, E or Ed395 were treated with Triton X-100; cell lysates and protein markers were subjected to 5 to 20% (wt/wt) sucrose gradient ultracentrifugation. Each of the 13 fractions was collected and subjected to Western blot analysis using a dengue-immune human serum [39]. (B, C) 5 to 20% sucrose gradient ultracentrifugation of lysates of 293T cells transfected with prMEd395 or Ed395. Each of the 14 fractions was collected and subjected to Western blot analysis [39]. Aliquots of fractions 14 and 6 were digested with endo H or PNGase F and subjected to Western blot analysis [39]. The size of molecular weight markers is shown in kDa. Arrow heads indicate Ed395 and the deglycosylated (dg) form.

We next analyzed the oligomeric status of E protein ectodomain, E and prM proteins by a sucrose gradient sedimentation analysis under non-denaturing condition [43], in which E protein ectodomain, E and/or prM proteins from transfected cells were solubilized with Triton X-100, a mild nonionic detergent to preserve the conformation of membrane protein. A control gradient of protein markers with known molecular weight was also performed as calibrations [43]. As shown in Figure 2A, the majority of E protein expressed by prME construct was found in the low-density fractions (fractions 6 to 8), which co-sedimented with the peak of prM protein (fractions 7 and 8), suggesting the formation of prM-E heterodimer. In contrast, the peak of prM protein (fractions 4 and 5) expressed by prMEd395 did not co-sediment with that of E protein ectodomain, which had two peaks (fractions 6 to 8 and fractions 12 to 13). Interestingly, E protein ectodomain expressed by Ed395 displayed a pattern similar to that of prMEd395, whereas E protein alone was found predominately in high density fractions (fractions 11 to 13). Comparing with the control gradient of protein markers, fractions 6 to 8 correspond to monomer and fractions 11 to 13 corresponds to tetramer or higher order complexes. Taken together, these findings suggest that in the absence of prM protein, E protein or E protein ectodomain alone can form large complexes, most likely E-E dimer or higher order complexes in high density fractions.

To rule out the formation of E aggregates in high density fractions (of mutants prMEd395 and Ed395), we repeated the sucrose gradient sedimentation analysis with 14 fractions, recovered the E protein from the highest fraction (fraction 14) and middle fraction (fraction 6, as a comparison), and conducted enzyme digestion with endo H and PNGaseF. As shown in Figures 2B and 2C, an endo H-resistant pattern was found for truncated E protein in fraction 14 (of both prMEd395 and Ed395), suggesting that the truncated E protein in fraction 14 has passed the quality control machinery in ER, transported beyond trans-Golgi, and therefore was unlikely to be aggregates retained in the ER or ER-associated degradation pathway [44].

### The E Protein Ectodomain Alone Associates with Membrane in Cells

**Figure 3:**
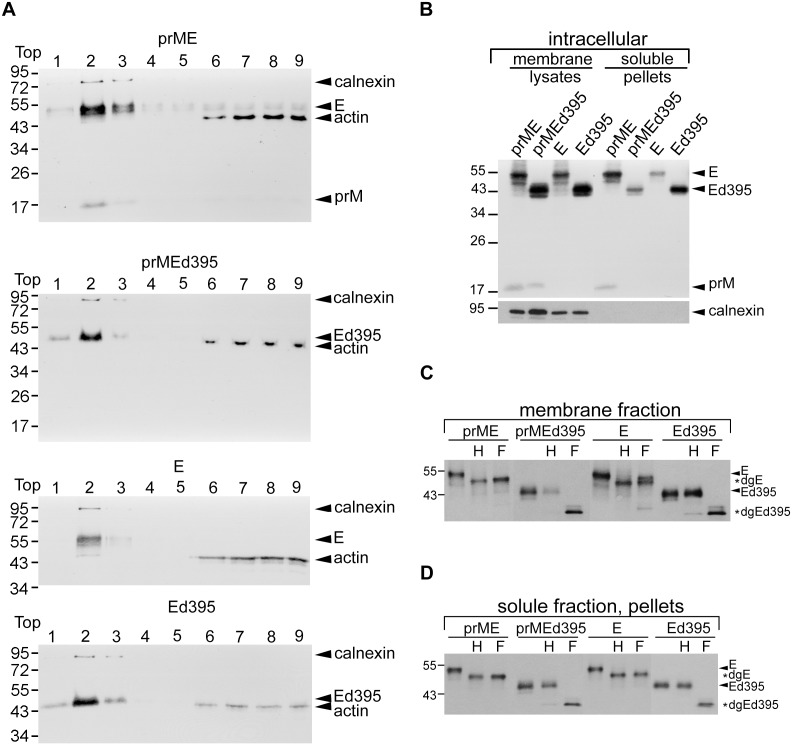
Membrane association of E protein and E protein ectodomain in the presence or absence of prM protein. (A) Membrane flotation assay. At 48 h post-transfection, 293T cells were homogenized and centrifuged to clear nuclei and debris. Supernatants were mixed with 72% sucrose, overlaid with 55% and 10% sucrose and ultracentrifuged; each of the 9 fractions was subjected to Western blot analysis using a dengue-immune human serum, anti-calnexin or anti-β-actin mAbs. (B) Subcellular fractionation assay. At 48 h, 293T cells transfected with prME, prMEd395, E or Ed395 were resuspended in modified buffer B and disrupted by freeze-thaw [38]. After clearing the nuclei and debris, membrane fraction and pellets derived from soluble fraction by sucrose cushion ultracentrifugation were subjected to Western blot analysis using a dengue-immune human serum (upper panel) and then reprobing with anti-calnexin mAb (lower panel). (C) Membrane fraction and (D) pellets of the soluble fraction of each transfectant were treated with endo H (H) or PNGase F (F), and subjected to Western blot analysis using a dengue-immune human serum. Arrow heads indicate E or Ed395, and aster indicates deglycosylated E (dgE) or deglycosylated Ed395 (dgEd395). One representative experiment of two was shown. The size of molecular weight markers is shown in kDa.

In the presence of transmembrane domains, flaviviral E protein has been shown to associate with membranes in cells [38], [45]. To examine if E protein ectodomain alone also associates with membrane, we carried out a membrane flotation assay that separates the membrane and cytosolic fractions [46]. As shown in Figure 3A, E protein expressed by prME and E constructs was found predominately in the membrane fractions (fractions 2 and 3), as indicated by an ER membrane protein, calnexin. Interestingly, E protein ectodomain expressed by prMEd395 and Ed395 also co-floated with membrane fractions but not cytosolic fractions (as indicated by β-actin), suggesting that in the absence of transmembrane domains, E protein ectodomain associates with membrane.

To further study membrane association and intracellular localization, 293T cells transfected with each of the four constructs were subjected to subcellular fractionation to separate the membrane-bound and soluble fractions. In agreement with our previous report [38], E protein expressed by prME construct was found in both the membrane fraction and pellets of soluble fraction, which contain VLPs [25], [38], suggesting that significant amounts of VLPs were produced and present in the lumen of ER or other compartments (Figure 3B). In contrast, E protein expressed by E, prMEd395 or Ed395 was found predominantly in the membrane fraction with a relatively small amount in the pellets of soluble fraction, suggesting the importance of stem/transmembrane domains and prM-E interaction for efficient production of VLPs in the lumen of intracellular compartments (Figure 3B). As a control, calnexin, an integral ER membrane protein, was detected in the membrane fraction but not in the soluble fraction. To study the intracellular localization of E protein, we analyzed the glycosylation pattern by endo H and PNGase F digestion. In both membrane and soluble fractions, E protein expressed by prME and E constructs was sensitive to endo H (Figures. 3C and 3D), suggesting that it contains high mannose glycans and retains in a compartment prior to trans-Golgi, most likely ER. In contrast, E protein ectodomain expressed by prMEd395 and Ed395 constructs was resistant to endo H, suggesting that it contains complex glycans that are completely processed in trans-Golgi (Figures. 3C and 2D). Taken together, these findings suggest that E protein ectodomain associates with membrane in or beyond trans-Golgi probably due to the lack of the stem/transmembrane domains, which contain an ER retention signal as reported previously [47].

### E Protein Ectodomain Alone Secretes as Soluble Protein and Particles in Supernatants

**Figure 4:**
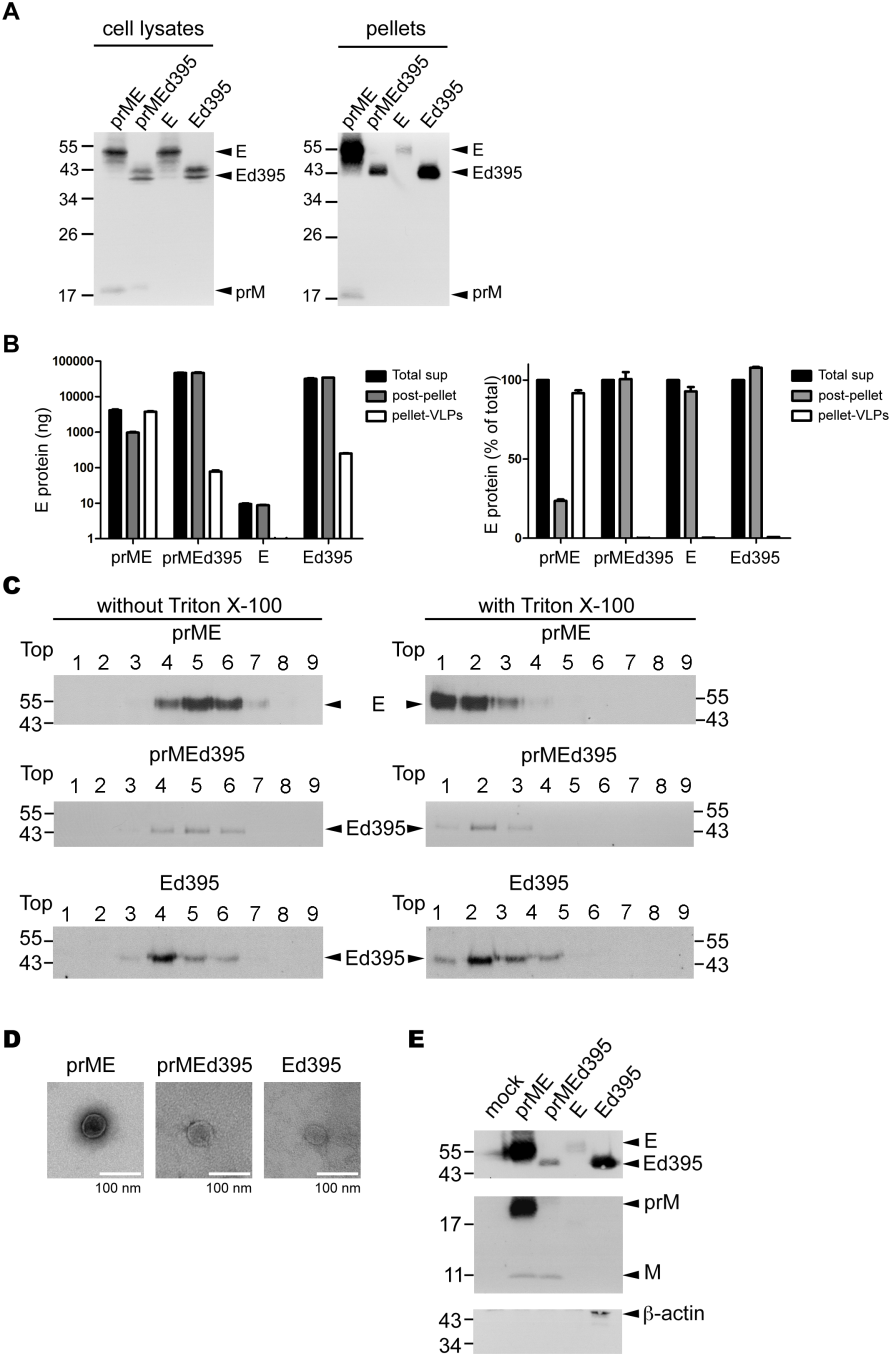
Production, detergent sensitivity and electron micrographs of VLPs. (A) At 48 h, cell lysates and pellets derived from culture supernatants of 293T cells transfected with prME, prMEd395, E or Ed395 were subjected to Western blot analysis using a dengue-immune serum [39]. (B) The amounts of various forms of E protein in culture supernatants, including total E protein in supernatants (Total sup), E protein in pellets containing VLPs (pellet-VLPs) and soluble E protein in supernatants post-ultracentrifugation (post-pellet) were quantified by a capture-ELISA as described in Methods. Right graph shows the proportion of E protein in pellet-VLPs and post-pellet (% of total E protein). (C) Pellets derived from culture supernatants of each transfectant were treated with or without 1% Triton X-100, followed by 15 to 55% (wt/wt) sucrose gradient ultracentrifugation; each fraction was subjected to Western blot analysis using a dengue-immune serum [39]. (D) An aliquot of the above pellets was stained with 2% PTA and photographed at the same magnification. (E) An aliquot of above pellets were subjected to Western blot analysis using a dengue-immune serum (top), rabbit anti-M serum (middle) and anti-β-actin mAb (bottom). The size of molecular weight markers is shown in kDa. One representative experiment of three was shown.

To further investigate whether E protein ectodomain can produce extracellular particles, pellets derived from culture supernatants, which contain VLPs [25], [38], were examined by Western blot analysis. Compared with that in the prME-transfected cells, the amounts of E protein in pellets relative to cell lysates in the prMEd395-, E- or Ed395-transfected 293T cells were severely reduced; this is in agreement with previous reports of the importance of stem/transmembrane domains in the production of VLPs (Figure 4A) [25], [38]. Of note, the amounts of E protein in pellets derived from Ed395-transfected cells was greater than that derived from prMEd395-transfected cells (Figure 4A).

We next performed a quantitative ELISA (Figure S1) to measure the amounts of total E protein in supernatants, E protein in pellets containing VLPs and soluble E protein in supernatants after ultracentrifugation, and their proportion. As shown in Figure 4B, the majority (more than 90%) of E protein expressed by prME construct was present in the pellets containing VLPs. In contrast, the majority of E protein expressed by prMEd395 and Ed395 was present in soluble E protein and less than 1% was in the pellets, probably forming VLPs. Very little amounts of E protein derived from E construct were found in supernatants; the possibility that they were derived from cell debris after transfection cannot be ruled out.

To exclude the possibility of protein aggregates in pellets derived from prMEd395 and Ed395, pellets were treated with or without 1% Trion X-100 and subjected to sucrose gradient ultracentrifugation. As shown in Figure 4C, in the absence of Triton X-100 pellets derived from 293T cells transfected with prME, prMEd395 or Ed395 sedimented as a discrete population (fractions 4 to 6) with a density of 1.13–1.15 g/cm^3^ (Figure S2), which is similar to that of flaviviral VLPs reported previously [48]. On the contrary, in the presence of Trion X-100 pellets derived from these three transfectants were solubilized and migrated to top fractions, suggesting that the pellets were in particulate form containing membrane rather than protein aggregates (Figure 4C). Further examination by EM revealed VLPs in the pellets derived from the three constructs (Figure 4D). Recently, cytoskeleton-driven mechanism has been shown to be involved in the budding of several RNA viruses [49]–[51]. To investigate whether cytoskeleton is involved in the formation of VLPs, we tested the incorporation of a cytoskeleton protein, β-actin, into VLPs. As shown in Figure 4E, β-actin but not β-tubulin (data not shown) was incorporated into the VLPs of Ed395, whereas neither was incorporated into the VLPs of prME or prMEd395. These findings suggest that β-actin is involved in the formation of VLPs derived from Ed395.

**Figure 5:**
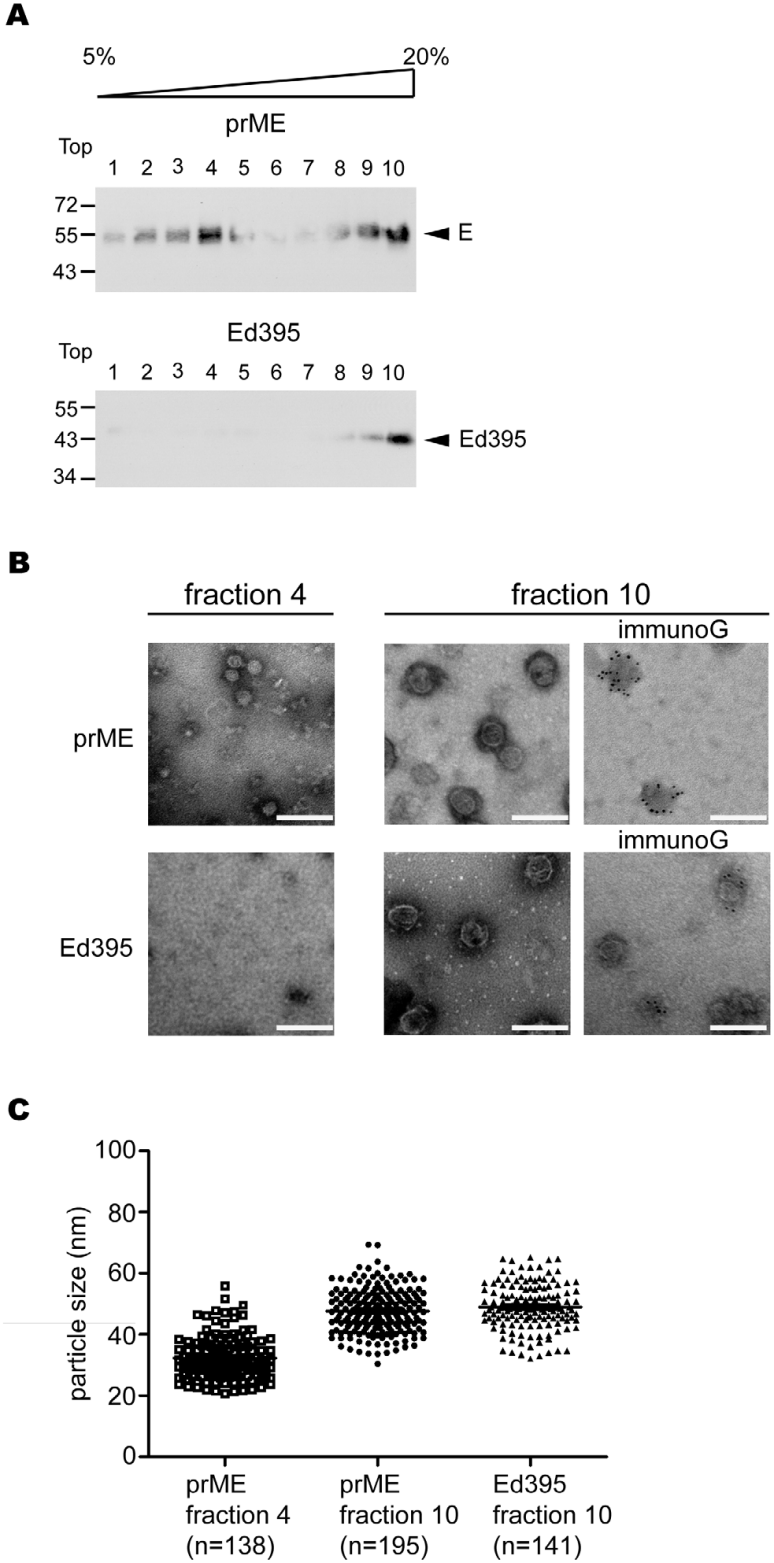
Sucrose gradient analysis and electron micrographs of purified VLPs. Pellets derived from 293T cells transfected with prME or Ed395 were subjected to a 5 to 20% (wt/wt) sucrose gradient ultracentrifugation; each of the 10 fractions was subjected to Western blot analysis using a dengue-immune serum. (B) Gradient-purified particles from fractions 4 and 10 were subjected to immunogold labeling using anti-E mAb (1H10-6-7), stained with 2% PTA and photographed at the same magnification. White bar: 100 nm. (C) The size of particles (diameter) of fractions 4 and 10 derived from prME and fraction 10 derived from Ed395 were determined by measuring more than 130 particles from electron micrographs. The particles size (diameter) is shown in nm.

To further examine the VLPs derived from Ed395 in comparison with those derived from WT prME, pellets containing VLPs were subjected to 5–20% sucrose gradient followed by Western blot analysis of each fraction [52]. As shown in Figure 5A, the majority of E protein in pellets derived from prME was detected in both top fractions (1 to 4) and bottom fractions (8 to 10), suggesting two major populations of VLPs with different sizes (Figure 5C). In contrast, E protein in pellets derived from Ed395 was only detected in the bottom fractions (8 to 10). The gradient-purified VLPs were further examined by EM including negative staining and immunogold labeling (Figures 5B and S3). The VLPs derived from prME had two sizes, small ones with a mean diameter of 32.3 nm (20.7 to 55.8 nm, fraction 4) and large ones with a mean diameter of 47.6 nm (30.4 to 69.4 nm, fraction 10) (Figure 5C), whereas the VLPs derived from Ed395 had a mean diameter of 49.0 nm (32 to 65.2 nm, fraction 10), similar to that of large VLPs derived from prME.

### N-linked Glycosylaiton Affect the Secretion of E Protein Ectodomain

**Figure 6:**
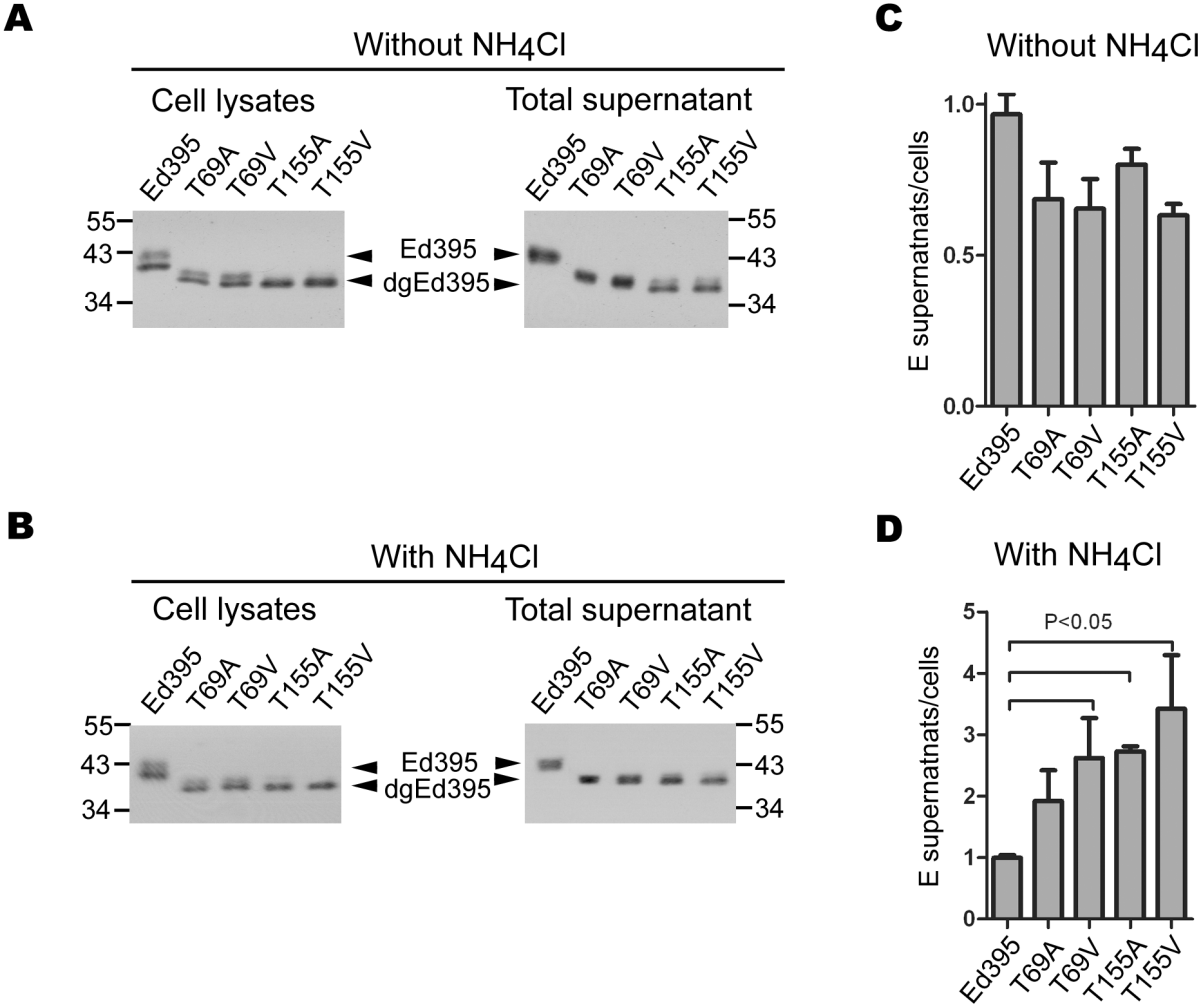
The effects of N-linked glycan and treatment with ammonia chloride on the secretion of E protein ectodomain. (A) At 48 h, cell lysates and culture supernatants of 293T cells transfected with Ed395 or each N-linked glycosylation mutant were subjected to Western blot analysis using a dengue-immune human serum. (B) At 16 h, transfected 293T cells were replaced with fresh medium containing 20 mM NH_4_Cl. At 48 h, cell lysates and culture supernatants were subjected to Western blot analysis. (C, D) The ratio of Ed395 in supernatants to Ed395 in cells without (C) or with (D) NH_4_Cl treatment. The amounts of Ed395 in cells and supernatants were quantified by capture-ELISA.

Previous studies have shown that mutations introduced to the N-linked glycosylation sites at residues 67 or 153 of E protein affected the release and infectivity of DENV virions [53]–[55]. These two mutations also reduced the secretion of VLPs [54]. To examine whether N-linked glycosylation affects the secretion of E protein ectodomain, site-direct mutagenesis was carried out to replace each of the two threonines (residues 69 and 155) at the third position of N-glycosylation sites with an alanine or valine in the Ed395 construct. Substitution introduced to residue at the third rather than first position of N-glycosylation site was employed, because previous study involving substitutions of residues at first (asparagine, residues 67 and 153) and third positions suggested the asparagine residue rather than N-linked glycan alone also affects assembly/release and infectivity of DENV in vitro [55]. Consistent with the loss of N-linked glycan, all 4 mutant E proteins migrated faster than the WT glycosylated E protein ectodomain (Figure 6A); the amount of E protein ectodomain expressed by the 4 mutants in cell lysates was comparable to that by WT Ed395 construct, suggesting that substitutions of alanine or valine do not affect the expression of E protein ectodomain. In contrast, the amount of mutant E protein ectodomain in supernatants compared to that of WT was slightly or moderately reduced (Figures 6A and 6C). After treating with ammonia chloride, which can increase the pH of the endosomal compartment, the amount of WT E protein ectodomain in supernatants decreased slightly, probably due to the effect of ammonia chloride on secretion. Interestingly, the amount of mutant E protein ectodomain in supernatants increased compared to that of WT (Figures 6B and 6D). Taken together, these findings suggest that alanine or valine substitution introduced to either threonine residue at the third position of two N-glycosylation sites reduce the secretion of E protein ectodomain, which can be restored by treatment with ammonia chloride.

## Discussion

In this study we investigated the DENV4 E protein ectodomain expressed in the presence or absence of prM protein. In the absence of prM protein, E protein ectodomain expresses well. Subcellular fractionation and enzyme digestion experiments revealed that E protein ectodomain associated with membrane in a compartment beyond trans-Golgi, whereas full-length E protein associated with ER membrane. In the absence of prM protein, E protein ectodomain can secrete as well as form particles, average 49 nm in diameter, based on sucrose gradient analysis and EM examination with immunogold labeling. To our knowledge, this is the first study reporting that E protein ectodomain alone can form particles. Furthermore, mutational analysis showed that removal of either N-linked glycosylation at residue 67 or 153 reduced the secretion of E protein ectodomain, which can be restored by ammonia chloride treatment. These findings suggest that N-linked glycan affects the secretion of E protein ectodomain.

Flaviviral E protein, a type I membrane protein, has been shown to associate with membrane in the cells, most likely through its two transmembrane domains and the stem region partially buried in membrane [38], [45]. Our findings that E protein ectodomain expressed by prMEd395 or Ed395, both lacking the stem and transmembrane domains, associated with membrane was unexpected. A crystallographic study of recombinant prM-E proteins complex revealed the heterodimeric interaction between the N-terminus of prM protein (mainly pr peptide) and E protein ectodomain at high resolution [56]. It is possible that E protein ectodomain expressed by prMEd395 interacts with N-terminus of prM protein (with two transmembrane domains), which contributes to the membrane association. Recent cryo-EM studies of mature DENV2 virions and mature and immature DENV1 virions at high resolution revealed that the N-terminus of M protein interacts with several hydrophobic residues in the helix αB of domain II facing the viral membrane [57], [58]. It is conceivable that in the absence of prM protein, these hydrophobic domain II residues of the E protein ectodomain expressed by Ed395 might interact with ER membrane during biosynthesis and contribute to the membrane association.

Previous studies of flaviviral VLPs including TBEV and WNV have reported the heterogeneity of VLPs in size with predominantly two populations, large and small VLPs [17], [41], [52]. In agreement with this, we run a similar 5–20% sucrose gradient and found two populations of VLPs (average 32.3 nm and 47.6 nm in diameter) expressed by DENV4 prME construct. In the study of TBEV VLPs, small VLPs were mainly found in WT prME construct (which generally cleaves prM efficiently), whereas both large and small VLPs were found in prM cleavage-deficient mutant, suggesting that the extent of prM cleavage may affect the size of VLPs [41]. Analysis of the recognition of large immature, small immature, large mature and small mature VLPs by 18 anti-E mAbs revealed that the maturation status rather than the size affected the antigenicity [41]. Since the prM cleavage of DENV has been shown to be inefficient [11]–[15], this may account for the almost equal proportion of large and small VLPs expressed by our DENV4 prME construct. Whether the size of DENV VLPs affects the recognition by different anti-E mAbs remains to be investigated.

It has been shown that transmembrane domains and prM-E interaction are critical for the production of TBEV and YFV VLPs [19], [25]–[27]. Our findings that DENV E protein ectodomain alone can produce VLPs, though a small proportion, was surprising. The presence of β-actin in the VLPs formed by Ed395 but not in the VLPs formed by WT prME construct, the relative homogeneity in size (average 49.0 nm in diameter) compared with the large and small VLPs formed by prME construct, and the intracellular localization of E protein ectodomain in a compartment beyond tran-Golgi compared with WT prM/E proteins in ER suggest that a different mechanism probably involving cytoskeleton-driven force to produce VLPs by Ed395. The molecular mechanism remains to be further investigated.

Previous studies have reported that mutations introduced to the N-linked glycosylation site at residues 67 and 153 of DENV2 E protein had severe and moderate effect, respectively, on the release and infectivity in mammalian cells and mild or no effect in mosquito cells [53]–[55]. These two mutations reduced the secretion of DENV2 VLPs; similarly mutations introduced to the N-linked glycosylation site at residue154 (the only N-linked glycosylation site) of E protein of linage I WNV and TBEV have also been shown to reduce the secretion of VLPs [48], [59]. Although altered interaction between E protein and chaperons in ER by removal of N-linked glycan at residue 67 and protein folding have been suggested to account for the impairment in secretion of DENV2 and VLPs [54], the mechanism remains unknown. Structural analysis of DENV E protein ectodomain dimer revealed that the N-linked glycan at residue 153 covers the fusion loop of domain II and two N-linked glycans can stabilize E-E dimer [22], [23]. Mutant DENV2, DENV3 and WNV with loss of N-linked glycan at residue 153 (or its equivalent) were found to fuse with target membrane at elevated pH or have lower stability at mildly acidic pH [6], [60], [61]. In this regard, our findings that reduced secretion of E protein ectodomain by removal of N-linked glycosylation at residue 67 or 153 could be restored by treatment with ammonia chloride suggest N-linked glycosylation at residue 67 or 153 of E protein ectodomain may help passing through acidic environment in the secretary pathway by stabilizing the E-E dimmer or preventing premature fusion with endosomal membrane.

We have also examined whether the conformation of secreted E protein ectodomain is similar to that of E protein on particles by immunoprecipitation assay with two mouse mAbs and human serum (Table S1). The amount of E protein ectodomain (expressed by prMEd395 or Ed395 construct) immunoprecipitated was generally comparable to that of E protein on VLPs (expressed by prME construct), suggesting that secreted E protein ectodomain preserves the epitopes recognized by these two mouse mAbs and human serum (Figure S4A). We also tested the conformation of secreted E protein ectodomain from different sources by a capture ELISA assay using 9 human mAbs with different epitopes/binding domains (Table S1) [36], [62]. Secreted E protein ectodomains prepared from 293T cells in the presence (prMEd395) or absence (Ed395) of prM protein or from Drodophila Schneicher 2 (S2) cells (recombinant E) [63] can be recognized similarly well by all 9 human mAbs tested (Figure S4B), suggesting that lacking prM protein does not affect the overall conformation of E protein ectodomain and that secreted E protein ectodomains derived form mammalian cells and insect cells preserve the epitopes recognized by all mouse and human mAbs tested. These findings are in agreement with our previous report [36] and suggest that the E protein ectodomain may be an ideal immunogen of subunit dengue vaccine, mimicking the native conformation of E protein without inducing anti-prM responses. To extend these observations, future studies to construct Ed395 of other serotypes and characterize their properties are needed. In addition, developing methods or optimizing conditions such as lowering the temperature to improve the biogenesis of Ed395 VLPs is warranted [64]. Moreover, studies to evaluate the immunogenicity including neutralizing antibodies induced by secreted Ed395 in animal models is critical for demonstrating Ed395 as a potential vaccine design. In this regard, it is worth noting that recombinant soluble E protein (corresponding to our Ed395) produced in Drosophila S2 cells can elicit neutralizing antibodies in mice and monkeys [63]. A recent study reported that Venezuelan encephalitis virus replicon particles expressing soluble E protein can induce neutralizing antibodies and provide protection in monkeys [65]. It is likely that soluble E protein and its VLPs produced by Ed395 construct may induce stronger immune responses than soluble E protein alone.

## Supporting Information

Figure S1 **Measurement of [E] in supernatants and pellets derived from culture supernatants of 293T cells transfected with prME, prMEd395, E or Ed395 construct by qELISA.** (A) Known concentration of recombinant DENV4 E protein (serial dilutions) were added to 96-well plate pre-coated with a human dengue-immune serum, followed by adding mouse anti-E mAb (1H10-6-7) and secondary antibody to generate a standard curve. (B) The OD450 of recombinant DENV4 E protein (left) and E protein in culture supernatants, including total E protein in supernatants (Total sup), E protein in pellets containing VLPs (pellet-VLPs) and soluble E protein in supernatants post-ultracentrifugation (post-pellet) were read. The [E] was determined by interpolating the OD value to the standard curve. Data are means with standard deviation of duplicates from one representative experiment of two.
(TIF)

Figure S2 **Detergent sensitivity and buoyant density of particles derived from culture supernatants of 293T cells transfected with prME, prMEd395 or Ed395 construct.** Pellets derived from culture supernatants of 293T cells transfected with prME, prMEd395 or Ed395 were treated with or without 1% Triton X-100, followed by 15 to 55% (wt/wt) sucrose gradient ultracentrifugation; each fraction was subjected to Western blot analysis using a dengue-immune serum [39]. The intensities of E band in each fraction were determined and presented as the percentage of total intensities of E band. One representative experiment of three is shown. The size of molecular weight markers is shown in kDa.
(TIF)

Figure S3 **Electron micrographs of purified VLPs from sucrose gradient.** Pellets derived from 293T cells transfected with prME or Ed395 were subjected to a 5 to 20% (wt/wt) sucrose gradient ultracentrifugation as in Figure 5. Gradient-purified particles from fractions 10 were stained with 2% PTA and photographed at low magnification (A) or subjected to immunogold labeling using anti-E mAb (1H10-6-7), stained with 2% PTA and photographed at high magnification (B).
(TIF)

Figure S4 **Recognition of secreted E protein ectodomain by mAbs and human serum using immunoprecipitation assay and capture-ELISA.** (A) Culture supernatants derived from 293T cells transfected with prME, prMEd395, E or Ed395 construct and recombinant DENV4 E protein ectodomain were immunoprecipitated using two mouse anti-E mAbs (4G2 and 1H10-6-7) and a confirmed dengue-immune serum (#13), and subjected to Western blot analysis. Anti-CD4 mAb is a negative control. (B) Culture supernatants derived from 293T cells transfected with prMEd395 or Ed395 construct and recombinant DENV4 E protein ectodomain were subjected to capture-ELISA using 9 human mAbs.
(TIF)

Table S1 **Summary of the E domains of epitope residues recognized by mAbs in this study.**
(DOC)
