# Supplementary figures and images for: Characterization of the Ectodomain of the Envelope Protein of Dengue Virus Type 4: Expression, Membrane Association, Secretion and Particle Formation in the Absence of Precursor Membrane Protein
Source: PLoS One. 2014 Jun 20;9(6):e100641. doi: 10.1371/journal.pone.0100641 (PMC4065094; doi:10.1371/journal.pone.0100641)

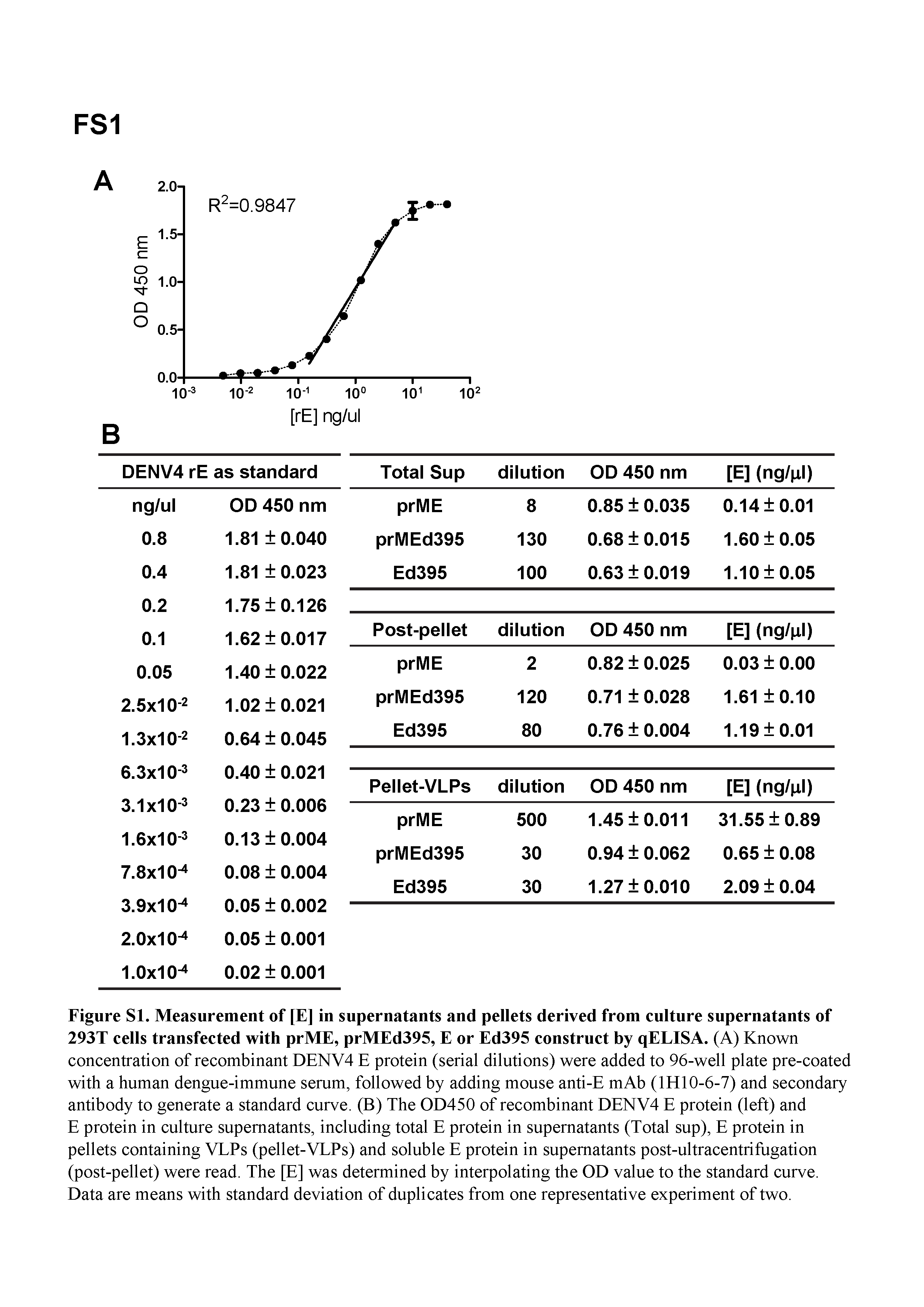

Supplement: Figure S1 — Measurement of [E] in supernatants and pellets derived from culture supernatants of 293T cells transfected with prME, prMEd395, E or Ed395 construct by qELISA. (A) Known concentration of recombinant DENV4 E protein (serial dilutions) were added to 96-well plate pre-coated with a human dengue-immune serum, followed by adding mouse anti-E mAb (1H10-6-7) and secondary antibody to generate a standard curve. (B) The OD450 of recombinant DENV4 E protein (left) and E protein in culture supernatants, including total E protein in supernatants (Total sup), E protein in pellets containing VLPs (pellet-VLPs) and soluble E protein in supernatants post-ultracentrifugation (post-pellet) were read. The [E] was determined by interpolating the OD value to the standard curve. Data are means with standard deviation of duplicates from one representative experiment of two. (TIF) [file pone.0100641.s001.tif]

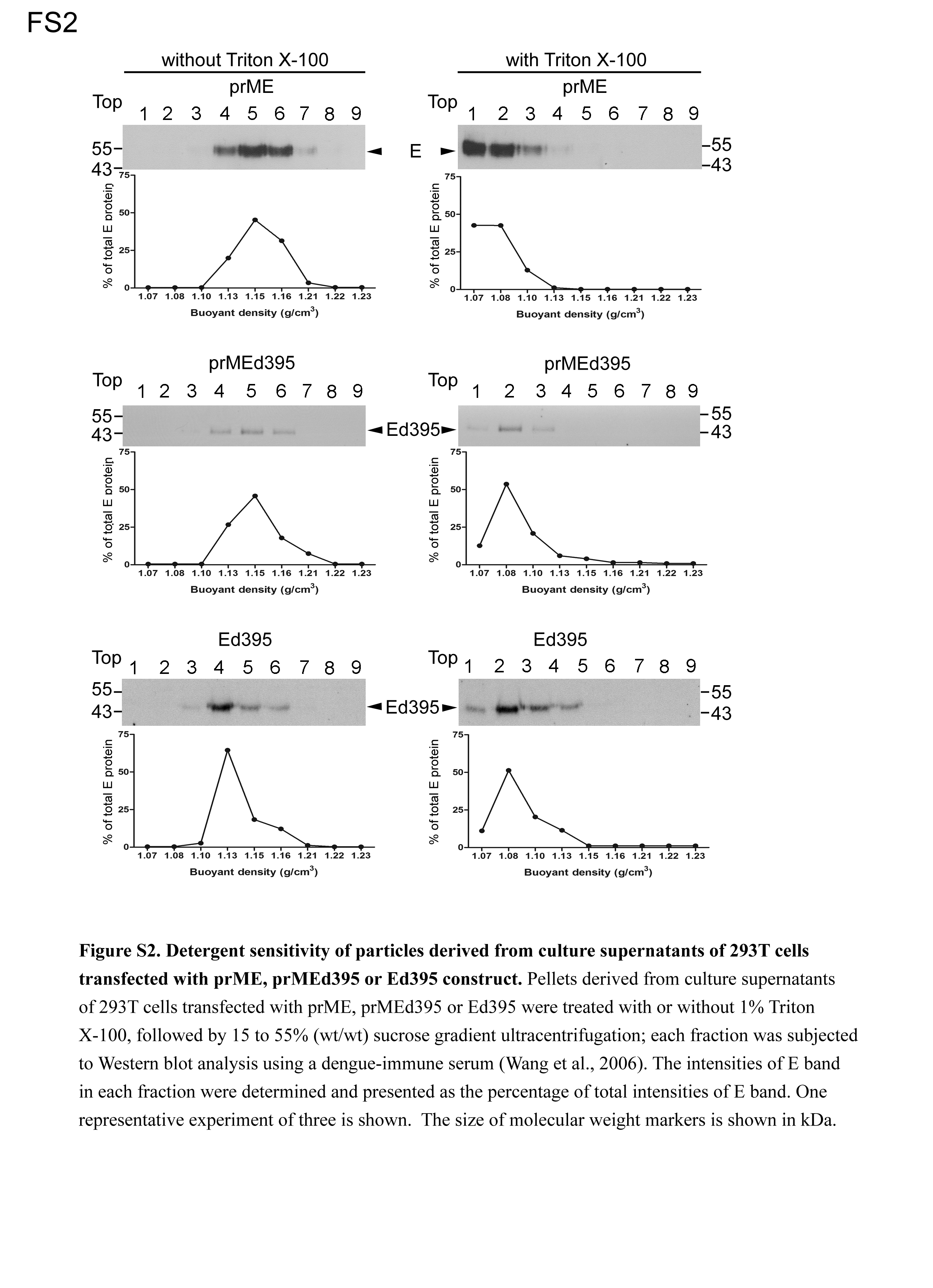

Supplement: Figure S2 — Detergent sensitivity and buoyant density of particles derived from culture supernatants of 293T cells transfected with prME, prMEd395 or Ed395 construct. Pellets derived from culture supernatants of 293T cells transfected with prME, prMEd395 or Ed395 were treated with or without 1% Triton X-100, followed by 15 to 55% (wt/wt) sucrose gradient ultracentrifugation; each fraction was subjected to Western blot analysis using a dengue-immune serum [39]. The intensities of E band in each fraction were determined and presented as the percentage of total intensities of E band. One representative experiment of three is shown. The size of molecular weight markers is shown in kDa. (TIF) [file pone.0100641.s002.tif]

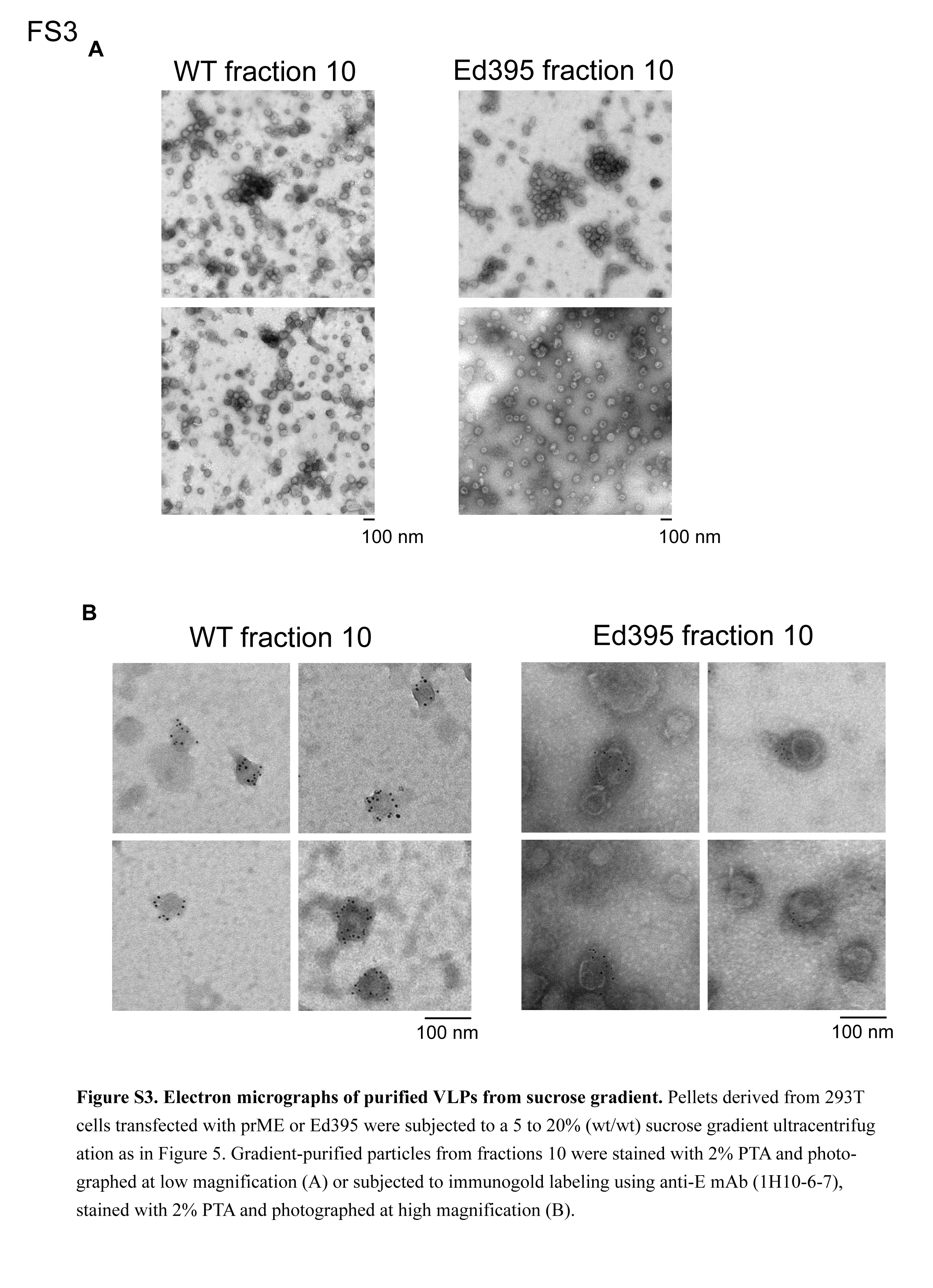

Supplement: Figure S3 — Electron micrographs of purified VLPs from sucrose gradient. Pellets derived from 293T cells transfected with prME or Ed395 were subjected to a 5 to 20% (wt/wt) sucrose gradient ultracentrifugation as in Figure 5. Gradient-purified particles from fractions 10 were stained with 2% PTA and photographed at low magnification (A) or subjected to immunogold labeling using anti-E mAb (1H10-6-7), stained with 2% PTA and photographed at high magnification (B). (TIF) [file pone.0100641.s003.tif]

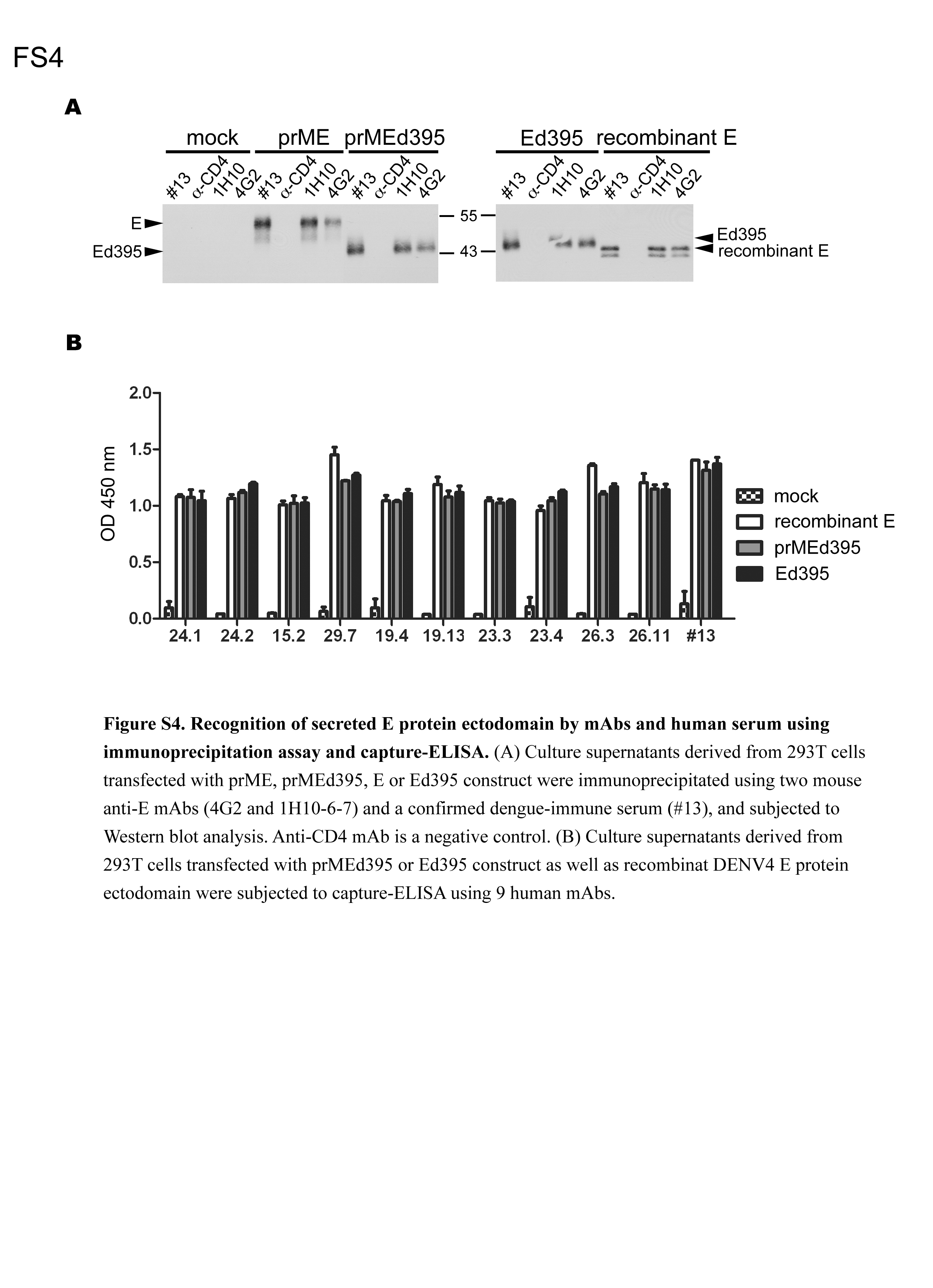

Supplement: Figure S4 — Recognition of secreted E protein ectodomain by mAbs and human serum using immunoprecipitation assay and capture-ELISA. (A) Culture supernatants derived from 293T cells transfected with prME, prMEd395, E or Ed395 construct and recombinant DENV4 E protein ectodomain were immunoprecipitated using two mouse anti-E mAbs (4G2 and 1H10-6-7) and a confirmed dengue-immune serum (#13), and subjected to Western blot analysis. Anti-CD4 mAb is a negative control. (B) Culture supernatants derived from 293T cells transfected with prMEd395 or Ed395 construct and recombinant DENV4 E protein ectodomain were subjected to capture-ELISA using 9 human mAbs. (TIF) [file pone.0100641.s004.tif]
