# Supplementary material for: Characterization of the Ectodomain of the Envelope Protein of Dengue Virus Type 4: Expression, Membrane Association, Secretion and Particle Formation in the Absence of Precursor Membrane Protein
Source: PLoS One. 2014 Jun 20;9(6):e100641. doi: 10.1371/journal.pone.0100641 (PMC4065094; doi:10.1371/journal.pone.0100641)
Supplement: Table S1 — Summary of the E domains of epitope residues recognized by mAbs in this study. (DOC) [file pone.0100641.s005.doc]

Table S1. Summary of the E domains of epitope residues recognized by mAbs in this study

| mAbs | Origin | Category*^a^* | E domain of epitope residues*^b^* |
| --- | --- | --- | --- |
| 4G2 | mouse | GR | domain II |
| 1H10 | mouse | TS | domain III |
| 24.1 | human | TS | domain II |
| 24.2 | human | TS | domain II |
| 15.2 | human | CR | domain II |
| 29.7 | human | CR | domain II |
| 19.4 | human | GR | domain II |
| 19.13 | human | GR | domain II |
| 23.3 | human | GR | domain II |
| 23.4 | human | GR | domain II |
| 26.3 | human | GR | domain II |

*^a^* GR: group-reactive; CR: complex-reactive; TS: type-specific to DENV4.

*^b^* Epitope residues were identified by a dot blot assay with a panel of 67 alanine mutants of predicted surface-exposed E residues and VLP-capture ELISA as described previously [62]; the E domains where the epitope residues were located are shown.
